# Heterogeneous decrease in malaria prevalence in children over a six-year period in south-western Uganda

**DOI:** 10.1186/1475-2875-10-132

**Published:** 2011-05-18

**Authors:** Pierre De Beaudrap, Carolyn Nabasumba, Francesco Grandesso, Eleanor Turyakira, Birgit Schramm, Yap Boum, Jean-François Etard

**Affiliations:** 1 Epicentre Mbarara Research Base, Mbarara, Uganda; 2 UMI 233, Institut de Recherche pour le Développement (IRD), Université Montpellier 1, Montpellier, France; 3 Epicentre, Paris, France; 4 Mbarara University of Science and Technology, Mbarara, Uganda

## Abstract

**Background:**

Malaria is a major public health problem, especially for children. However, recent reports suggest a decline in the malaria burden. The aim of this study was to assess the change in the prevalence of malaria infection among children below five years of age between 2004 and 2010 in a mesoendemic area of Uganda and to analyse the risk factors of malaria infection.

**Methods:**

Two cross-sectional surveys were conducted in 2004 and in 2010 at the end of the rainy and dry seasons to measure the prevalence of *P. falciparum *infection among children less than five years of age. Rapid diagnostic tests and blood smears were used to diagnose malaria infection. In 2010, sampling was stratified by urban and rural areas. In each selected household, knowledge of malaria and bed nets, and bed net ownership and use, were assessed.

**Results:**

In 2004 and 2010, respectively, a total of 527 and 2,320 (999 in the urban area and 1,321 in rural areas) children less than five years old were enrolled. Prevalence of malaria infection declined from 43% (95% CI: 34-52) in 2004, to 23% (95% CI: 17-30) in rural areas in 2010 and 3% (95% CI: 2-5) in the urban area in 2010. From the rainy to dry season in 2010, prevalence decreased from 23% to 10% (95% CI: 6-14) in rural areas (*P *= 0.001) and remained stable from 3% to 4% (95% CI: 1-7) in the urban area (*P *= 0.9). The proportion of households reporting ownership and use of at least one bed net increased from 22.9% in 2004 to 64.7% in the urban area and 44.5% in rural areas in 2010 (*P *< 0.001). In 2010, the risk of malaria infection was consistently associated with child age and household wealth. In rural areas, malaria infection was also associated with geographic factors.

**Conclusions:**

This study reports a significant drop in the prevalence of malaria infection among children below five years of age, paralleled by an uptake in bed-net use. However, prevalence remains unacceptably high in rural areas and is strongly associated with poverty.

## Background

Malaria is still a major public health problem in sub-Saharan Africa, where 85% of cases and almost 90% of malaria-related deaths occur [1,2]. However, recent epidemiological reports indicate significant decreases in malaria prevalence in several areas [3-11], yet it remains unacceptably high in other areas [11-14]. In Uganda, malaria is highly endemic and is ranked among the first causes of morbidity and mortality affecting especially young children [15,16]. As the heterogeneity of malaria transmission is considerable, epidemiological findings vary greatly between areas (e.g, urban versus rural), and additional data are required to better understand the malaria situation [14,16-18].

These epidemiological changes were observed after several interventions to combat malaria [19], including the use of artemisinin-based combination therapy (ACT), distribution of insecticide-treated bed nets (ITNs), indoor residual spraying (IRS), intermittent preventive treatment of pregnant women (IPTp), and more recently use of malaria rapid diagnostic tests (RDTs) [19,20]. Although declines in malaria transmission generally occurred after implementation of these interventions, a direct causal link is difficult to establish [2,11].

A common challenge in analysing the effect of these interventions is considering a combination of interlinked factors instead of separate ones. A limitation of the available data is that they often come from routine surveillance systems, which do not necessarily reflect the situation in the community and usually rely on clinical presumptive diagnoses. Therefore, additional population-based data are needed to confirm observed surveillance trends and analyse the observed heterogeneity in malaria prevalence changes, as well as to document the actual impact of the various interventions. For malaria control and eradication, a better understanding of the factors associated with malaria prevention is important to enhance the quality of interventions and inform public health decisions.

The situation is even more complex for children aged less than five years. Young children pay a heavy price for malaria infection, accounting for nearly 88% of deaths related to malaria in sub-Saharan Africa. However, the heterogeneity of the risk of malaria infection and fatal outcome are the highest for this population and not fully understood [21].

This study presents the results of two cross-sectional surveys done at a six-year interval in a rural area of south-western Uganda. The first cross-sectional survey was conducted in 2004 to measure the prevalence of confirmed infection with *Plasmodium falciparum *in the general population. The second survey was conducted in 2010 as part of a wider global study aiming to collect epidemiological data on *P. falciparum *infection among children aged less than five years; gather information on knowledge, perception, and behaviours related to malaria; and assess the performance of various diagnostic methods. This report focuses on the changes in the epidemiology of *P. falciparum *infection in children aged less than five years over a six-year period and on the analysis of associated risk factors.

## Methods

### Geographic area

This study was conducted in four districts located in south-western Uganda formally referred to as the Greater Mbarara district. The total population was estimated at 1,088,356 inhabitants in 2002, and the main town is Mbarara municipality with a population of 69,363 [22]. This area is mainly rural, lying at an altitude ranging between 130 and 1,500 m above sea level, with vegetation a mixture of bush and short grass. The climate is tropical with a bimodal rainfall pattern averaging 1,200 mm per annum in September-January and March-May. In Greater Mbarara district, malaria transmission is considered mesoendemic.

### Study design and population

Two cross-sectional surveys were conducted. The first survey was performed between mid-January and mid-February in 2004. The second survey was conducted in two parts: first round in mid-January to mid-February 2010 at the end of the rainy season; and second round in August 2010 at the end of the dry season.

In 2004, three-stage cluster sampling was used to select individuals to be included in the survey. A total of 30 parishes were sampled with probability proportional to the size. Within each cluster, one village was randomly selected, and within each village 30 households randomly selected using the EPI method [23,24]. All the persons usually living in the household were included in the survey, but only children aged less than five years were included in this analysis. A malaria RDT was performed for all study participants, and a blood smear taken for all positive RDTs.

In the first round of the 2010 survey (January), two-stage stratified cluster sampling was used to select households and the children within the households. Stratification was performed by classifying Greater Mbarara district into an urban area (Mbarara municipality) and rural area (remaining clusters). A total of 33 villages were randomly selected in each stratum with a probability proportional to their population size. Households were then randomly selected in each village using the EPI method, and all children less than five years of age from the households were included in the survey until a total number of 20 children per village was reached. For all children, RDT and blood smear were performed.

In the second round of the 2010 survey (August), the same 33 villages were selected for the rural stratum, whereas only 17 clusters were sampled from the urban stratum using the same method.

### Survey procedure

Household locations and altitudes were recorded using a global positioning system (GPS) receiver (eTrex, Garmin Ltd, Olathe, KS, USA). Standardized questionnaires were administered to the child's parent or legal representative by qualified medical personnel in their preferred language (English or local language). Demographic and socioeconomic data of each household were collected.

An indirect measure of the socio-economic status (SES) was derived from characteristics of the house (walls: mud versus bricks; roof: thatched versus iron sheet) and whether or not the household possessed the following assets: mobile phone, radio, television, motorbike, bicycle and electricity. From the factor analysis of the eight items, two distinct factors emerged. The first factor was made of the housing characteristics whereas the second factor of five out of the six asset indices. As loading factors were similar and close to one for each factor, two simple scores were eventually computed by summing the indicator variables and later referred as "housing score" (type of the walls + type of the roof) and "economic score" (sum of the responses whether the household possessed the assets) [25].

Knowledge of malaria and prevention strategies was assessed. Bed net ownership and actual use of bed nets were directly controlled by the investigator. Each child in the study received a medical examination, including height, weight, and temperature.

### Laboratory methods

#### Blood smear

Blood smear and RDT were carried out on the same finger-prick blood sample. A thin blood film for confirmation of *Plasmodium *species and a thick blood film for microscopic detection and parasite density count were prepared and stained with 10% Giemsa. Asexual parasites were counted until 200-500 leucocytes were counted and converted to the number of parasites per unit volume, assuming 8,000 leucocytes/μL of blood. Gametocytes were reported after examining 100 microscopic fields. Slides were considered negative if no parasites were detected after viewing 100 microscope fields.

In the 2010 survey, the slides were stained in the laboratory by a technician different from the one in the field and double-read following blinding. In case of discordant results between the two microscopists (defined as positive/negative discordance for asexual stages; species discordances for asexual stages; asexual parasite density discordance >50%; and gametocyte or malaria pigment discordance), a third reading was performed by the most experienced microscopist and taken as the definitive result. In the 2004 survey, the slides were read by only one microscopist.

#### Rapid diagnostic tests

The RDT used for both studies was Paracheck^® ^(Orchid Biomedical System, catalogue No 30301025, Verna, Goa, India). RDTs were stored in their original packaging in a room with monitored temperature. RDTs were performed by trained laboratory technicians. A test result was considered positive if both the internal control and the test band were stained; negative if only the internal control was stained; and invalid if the internal control was not stained.

### Statistical methods

Data were double entered using EpiData (version 3.1, Odense, Denmark). Statistical analysis was performed using the open software R (version 2.12.1, R Project for Statistical Computing; http://www.r-project.org/) [26].

#### Prevalence estimates and comparison

Prevalence of infection with *P. falciparum *was computed using positive RDT results corrected by blood smear for the 2004 survey, and both positive RDT results corrected by blood smear and blood smear only for the 2010 survey. Comparison between the prevalence estimates of 2004 and 2010 was done using RDT results corrected by blood smear for both surveys. Prevalence was computed separately for 2004 and each stratum (urban and rural) of 2010 using the Horvitz-Thompson estimator [27]. Variance of the estimates was computed using the linearization method [27]. Categorical data were compared using the chi-square test and continuous variables using the t-test or a non-parametric test when appropriate. All tests results were corrected for the clustered design.

Proportions of respondents with relatively high knowledge of malaria, bed-net ownership, and bed-net use were separately estimated for urban and rural strata in the 2010 survey. The relationship between malaria knowledge, bed net ownership and use, and household characteristics was assessed through logistic models with robust variance.

Associations between risk of malaria infection (assessed using blood smear-corrected RDT for 2004 and blood smear only for 2010) and the variables collected were analysed using generalized, multilevel, mixed-effects models with a logit link. Village size was included in all models to account for unequal selection probabilities proportional to village size. The variables assessed fell under three classifications: (1) individual-level variables: child's age, gender, weight (Z score), weight-for-height (Z score), and use of a bed net by the child during the night preceding the survey; (2) household-level variables: household size, wealth measured by the housing and economic scores, education level of the head of the household, bed net ownership, number of bed nets owned, bed-net use by the head of the household the night preceding the survey; and (3) village (cluster)-level variables: village size, bed net coverage, mean economic score, and for rural areas, mean altitude and geospatial coordinates (longitude and latitude standardized and centred around Mbarara municipality). In the 2004 survey, only child's age and gender, household size, housing score, education level of the household, village size, mean altitude and geospatial coordinates were collected and available for the analysis. In all models, unexplained non-spatial variation was accounted at the village (cluster) levels. As preliminary analysis indicated that the variance estimates in the rural and urban clusters were highly different, separate variance components were estimated for each survey

In all models, goodness of fit was checked using graphical assessment of the residuals and general diagnostics methods [28,29].

#### Ethics approval

Written informed consent for study participation was obtained from the children's parents or guardians. Children with a positive RDT result were immediately treated according to the Ugandan national malaria policy. The study was approved by the institutional review boards of Mbarara University of Science and Technology (MUST), Uganda National Council for Science and Technology, and France's Comité de protection des personnes - Ile-de-France XI.

## Results

### Population description

A total of 264 households were included in the 2004 survey, and 1,598 in the 2010 survey (Table 1). The first round of the 2010 survey included 941 households (490 urban, 451 rural), while the second round included 657 (235 urban, 422 rural). Less than 10% of the households contacted declined to participate to the surveys.

**Table 1:** Household and individual characteristics of study population, 2004 and 2010

Households characteristic		2010		2004
		Urban n = 725	Rural n = 873	n = 264
Mean household size (95% CI)		4.9 (4.7-5.0)	5.6 (5.4-5.7)	6.9 (6.0-7.9)
Mean number of children <5 years old (95% CI)		1.5 (1.5-1.6)	1.6 (1.5-1.7)	1.5 (1.4-1.6)
Education level^§^, % (95% CI)	None	4 (3-6)	18 (14-22)	14 (8-19)
	Primary	34 (27-42)	61 (57-65)	68 (62-74)
	Secondary	39 (32-46)	17 (13-20)	15 (11-20)
	Tertiary	22 (14-31)	4 (2-6)	3 (1-6)
Mean socioeconomic score^# ^(95% CI)		50.2 (47.5-53.0)	30.5 (28.0-32.9)	--
House structure, % (95% CI)	Wall type*	87 (82-91)	27 (20-34)	27 (16-37)
	Roof type^†^	99 (99-100)	89 (86-92)	84 (77-91)
**Individual characteristic**		**2010**		**2004**
		**Urban n = 999**	**Rural n = 1,321**	**n = 527**
Mean age, months (95% CI)		25.2 (24.0-26.5)	28.3 (27.4-29.1)	30.9 (29.3-32.6)
Mean weight-for-age, Z score (95% CI)		-0.34 (-0.49 to 0.19)	-0.86 (-1.00 to -0.71)	--
Mean height-for-age, Z score (95% CI)		-1.07 (-1.40 to -0.74)	-2.20 (-2.57 to -1.83)	--
Mean weight-for-height, Z score (95% CI)		0.39 (0.06-0.72)	0.69 (0.28-1.10)	--

^§^Highest education level attended by the head of the household; ^# ^Standardized score computed using data on household's ownership of various assets (see detail in Method section); *Wall made of brick *vs *mud; ^†^Roof made of iron sheet *vs *thatched

Characteristics of the households were different between rural and urban areas (Table 1). On average, higher education level, housing, and socioeconomic scores were observed in the urban area compared with rural areas (*P *< 0.001). However education level and housing scores were similar between households surveyed in 2004 and those in rural areas surveyed in 2010 (*P *= 0.4). As expected, occupations of the head of household differed between rural and urban areas, with rural heads of households more likely to be farmers compared with the urban area (54%, 95% CI: 45-62 in rural area vs 4%, 95% CI: 1-6 in urban).

In the 2004 survey, 527 subjects were aged less than 5 years and therefore included in this analysis (Table 1). In 2010, a total of 2,320 children aged less than 5 years were included, 999 in the urban area (661 and 338 in the first and second rounds, respectively) and 1,321 in rural areas (664 and 657 in the first and second rounds, respectively). Children from the rural areas were more likely stunted as shown by lower weight-for-age and height-for-age Z scores (*P *< 0.001).

### Changes in malaria epidemiology

In 2004, the prevalence of *P. falciparum *infection among children less than five years old was 43%, 95% CI: 34-52, based on RDT diagnosis corrected for false positives by blood smear. Compared with 2004, in 2010 at the end of the rainy season prevalence decreased significantly to 23%, 95% CI: 17-30 in rural areas and 3%, 95% CI: 2-5 in the urban area (*P *< 0.001).

The proportion of clusters with estimated prevalence >50% decreased from 43% in 2004 to 9% in rural areas and 0% in the urban area in 2010, whereas the proportion of clusters with estimated prevalence <1% increased from 20% in 2004 to 24% in rural areas and 70% in the urban area in 2010 (*P *< 0.001).

In the dry season in 2010, estimated malaria prevalence remained relatively low and stable in the urban area at 4%, 95% CI: 1-7, compared with 3%, 95%CI: 2-5 during the rainy season (*P *= 0.9). However, prevalence decreased significantly to 10%, 95% CI: 6-14 in rural areas, compared with 23%, 95%CI: 17-30 during the rainy season (*P *= 0.001).

**Figure 1:**
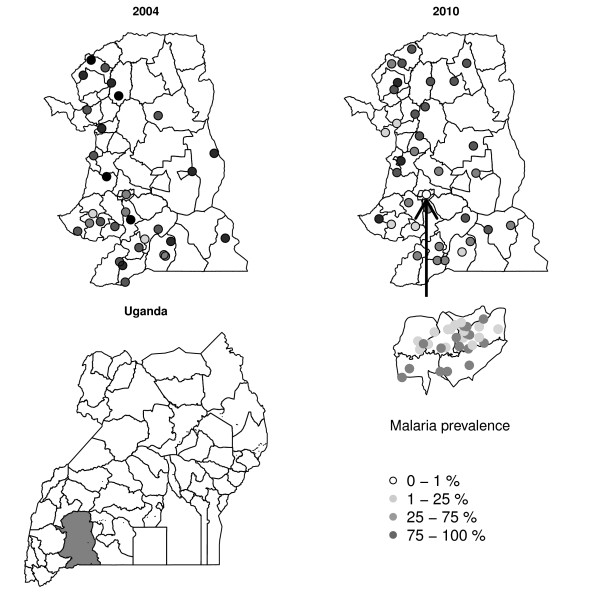
**Maps of Uganda and of Greater Mbarara district with the cluster-levels prevalence estimations of *P. falciparum *infection among children for 2004 (top left plot) and 2010 (top right plot)**. Circles indicate cluster locations. The inset plot shows in enlarged Mbarara municipality.

Compared with RDT diagnosis, the prevalence of malaria infection measured by blood smear in 2010 was 4%, 95% CI: 2-6, 4%, 95% CI: 1-6, 25%, 95% CI: 18-32, and 13%, 95% CI: 8-17, respectively, for the urban area during rainy season, urban area during dry season, rural areas during rainy season, and rural areas during dry season. The distribution of malaria prevalence during the rainy season in the survey villages in 2004 and 2010 show heterogeneity in terms of spatial structure in the rural areas (Figure 1).

### Malaria prevention: knowledge and behaviours

In terms of knowledge about malaria, most respondents mentioned mosquitoes as the vector of malaria, though less often in rural areas (77%, 95% CI: 70-83) compared with the urban area (96%, 95% CI: 93-98; *P *< 0.001). In adjusted analysis, other factors associated with knowledge of mosquitoes as the malaria vector were higher education level (OR = 2.58, 95% CI: 1.87-3.56) and higher socioeconomic level (OR = 1.29, 95% CI: 1.09-1.54).

When knowledge of malaria prevention methods was assessed, 96% (95% CI: 93-98) of those in the urban area and 76% (95% CI: 71-84) in rural areas mentioned the use of a bed net as a prevention strategy (*P *< 0.001). Knowledge of bed nets as a prevention method was also positively associated with knowledge of mosquitoes as the vector of malaria (OR = 16.31, 95% CI: 10.11-26.31) and socioeconomic level (OR = 1.37, 95% CI: 1.12-1.67).

Significantly more households in the urban area (78%, 95% CI: 72-85) reported owning at least one bed net compared with the rural areas (57%, 95% CI: 46-67; *P *< 0.001). However, after adjusting for education, socioeconomic level, and knowledge of prevention methods, this association weakened (See Additional file 1). Urban households more often purchased bed nets (87%, 95% CI: 81-92), while rural households more often received bed nets as donations (67%, 95% CI: 50-84). More than half of bed nets were treated with insecticide (51%, 95% CI: 37-63 in rural area, 69%, 95% CI: 56-83 in urban; *P *= 0.6).

The proportion of households reporting use of a bed net the night prior to the interview increased from 23%, 95% CI: 12-33 in 2004 to 65%, 95% CI: 58-72 in 2010 (first round/rainy season) in the urban area and 45%, 95% CI: 36-53 in rural areas (*P *< 0.001). A majority of the households owning at least one bed net reported its use by an adult in the night prior to the interview (86%, 95% CI: 81-91 in urban area, 79%, 95% CI: 72-86 in rural; *P *= 0.1), as well as by children (86%, 95% CI: 80-93 in urban area, 69%, 95% CI: 60-78 in rural; *P *= 0.002). Bed net use was more frequent amongst adults with higher education and socioeconomic levels (See Additional file 1). These factors, as well as household size and knowledge of prevention methods, were associated with the use of bed nets for children (See Additional file 1).

Of note, as a result of a massive donation of bed nets in 2010, the proportion of households owning a bed net increased further to 92%, 95% CI: 87-97 and 76%, 95% CI: 66-86 in the urban and rural areas, respectively, as assessed during the second round (dry season; August) of the 2010 survey.

### Factors associated with malaria prevalence

**Figure 2:**
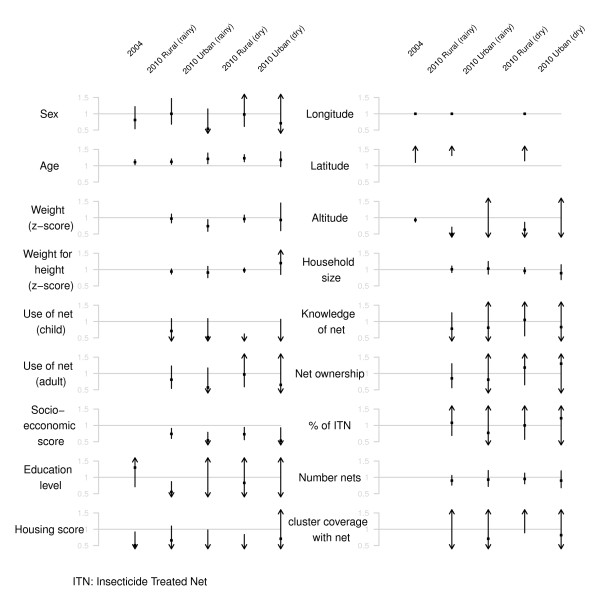
**Factors associated with malaria prevalence in children less than 5 years old across five study settings (2004, 2010 rainy rural, 2010 rainy urban, 2010 dry rural, 2010 dry urban)**. Point estimates of odds ratios with 95% confidence intervals are represented on the y-axis. Confidence intervals exceeding the graph scale are indicated with an arrow.

The association of *P falciparum *infection amongst children less than five years old and various individual, household, and village-level factors was assessed in univariate analyses in the 2004 survey and for each round and stratum (urban *vs *rural) in the 2010 survey. In univariate analysis, lower socioeconomic score of the household and higher age of the child were consistently associated with increased risk of malaria infection across the various settings (Figure 2). Longitude and altitude were also consistently associated with malaria risk but only in rural areas and in the 2004 survey (Figure 2). Interestingly, the later association was not linear with a clear threshold around 1,500 m. A consistent yet not significant association was seen between bed-net use by the child in the night preceding the survey and lower risk of malaria infection.

In adjusted analysis in 2010, younger age, higher housing and socioeconomic scores, and bed-net use by the child in the night preceding the survey were associated with lower risk of malaria infection without evidence of heterogeneity between the various settings (Table 2). In rural areas, geographic factors were also important predictors of malaria infection as indicated by negative associations with elevation (OR = 0.05, 95% CI: 0.01-0.23 for altitude >1,500 m compared with ≤1,500 m) and latitude (OR = 1.55, 95% CI: 1.17-2.05 per standard deviation). In the urban area but not in rural areas in 2010, higher weight-for-age Z score was associated with lower risk of malaria infection (OR = 0.80, 95% CI: 0.62-1.03 in the urban area; OR = 1.00, 95% CI: 0.92-1.09 in rural areas).

**Table 2:** Multivariate analysis of malaria prevalence in children <5 years old, stratified by residence area, 2010 (n = 1,325)

	Odds Ratio (95% CI)
Child age (per 6 months)	1.16 (1.10-1.23)
Weight-for-age (Z score)	
Urban area	0.80 (0.62-1.03)
Rural area	1.00 (0.92-1.09)
Housing score	0.61 (0.41-0.91)
Socioeconomic score*	0.75 (0.64-0.89)
Education level (head of household)	0.87 (0.60-1.26)
Latitude	
Urban area	0.72 (0.44-1.16)
Rural area	1.55 (1.17-2.05)
Altitude (≤1,500 vs >1,500 m)	0.05 (0.01-0.23)
Child slept under bed net	0.61 (0.44-0.83)

*Score reduced and standardized

No systematic spatial pattern was found in the model residuals as indicated by variogram (data not shown) and Moran index (*P *= 0.99). In sensitivity analysis, exclusion of clusters with an observed prevalence of zero did not alter the estimations, except for the effect of altitude in rural areas. This was further explored using logistic regression to assess the association of factors measured at cluster level with probability of a cluster having a prevalence equal to zero in rural areas. In adjusted analysis, altitude was the only significantly associated factor, with an increased probability of cluster prevalence equal to zero as altitude increased (OR = 5.12 per 100 m, 95% CI: 2.02-12.95).

## Discussion

This study documents an apparent decline in the prevalence of infection with *P. falciparum *in children less than five years old and an increase in the use of bed nets over a six-year period in Greater Mbarara district, a mesoendemic area of Uganda. Similar results have been reported in other settings but were often based on routine surveillance data [3-10]. In this study, the same area was observed before the implementation of the Roll Back Malaria strategy for malaria control and six years after, with data systematically collected at individual, household, and village levels, and malaria infections assessed using blood smear or RDT. These results are thus less likely to be confounded by ecological bias or by changes over time in the reporting system than reports from routine surveillance. Moreover, the high participation rate observed makes non-response bias very unlikely.

Despite these encouraging findings, the malaria situation remains of concern in the rural areas of this region, contrasting with the urban situation; some rural villages still have medium to high prevalence of *P. falciparum *infection, and almost 10% of the rural villages had a mean prevalence exceeding 50%. A parallel could be drawn with the global Ugandan malaria epidemiological situation that is characterized by areas of high malaria transmission contrasted to low malaria transmission in urban areas [14,16-18,30].

Also, despite improvements since 2004, the coverage of households with bed nets remained low in rural areas and below the target proposed by WHO [2]. Estimations of bed net coverage are commonly limited by the fact that they are based on reported information. In this study, actual presence of bed net was controlled in all households, thus the estimated bed net coverage is believed to be reliable and confirms the result from a recent national survey [30]. As reported in other studies, it was found that prevention strategies are different between rural and urban areas, mainly because of differences in the household wealth and education levels [31,32]. Further research is needed to better understand these differences and more generally the reasons for bed net use [33].

To identify factors associated with increased malaria risk, individual-, household-, and cluster-level characteristics were considered. At cluster/village level, an altitude above 1,500 m was a strong predictor of very low malaria prevalence. However, below 1,500 m, the association between altitude and risk of malaria infection vanished. Also at cluster level, a strong association between the malaria risk and latitude was observed that indicates the presence of unexplained heterogeneity with a spatial structure that should be further investigated.

At household level, higher wealth as measured by higher housing and socioeconomic scores was strongly associated with decreased risk of malaria infection in both rural and urban areas, consistent with other studies [34,35]. Wealth was an important factor associated with bed-net ownership. A possible explanation for the lower risk of malaria infection with increased wealth is that more expenses may be dedicated to prevention in wealthier households. A complex association between household characteristics (wealth, education level, and size) on one hand, and knowledge, ownership, and actual use of bed nets on the other was found, which could explain the weak association between bed-net use and malaria infection. The difference in the origin of the nets between rural and urban settings suggest that net donation remains an important way to improve the net coverage. However, as indicated by the moderate use of net utilization by children, bed-net donations should be coupled with health education.

At individual level, the risk of malaria increased with the age of the children in both rural and urban areas. A possible explanation of this is that younger children may be more likely to stay under bed nets as they usually sleep with their mothers and play less in the evenings. Although the association did not reach statistical significance in the separate analyses, in the pooled analysis malaria risk was lower for children reported to have slept under a bed net the night preceding the survey interview.

This study has several limitations. First, accurate information on malaria interventions in the study area over the study period (especially on ACT availability) was not collected. In 2004, the information collected was less comprehensive and the diagnosis method was less accurate, compared with 2010. Although the same area was investigated, the cross sectional design of this study leads to limitations that include the inability to account for short term parasitemia fluctuations. Also, it should be noticed that malaria risk is known to vary on very small scale, thus only a rough estimation of the spatial variation of malaria could be done with the sampling design used. This study focused on children less than five years of age, since they constitute the most vulnerable population for malaria morbidity and mortality. However, as a consequence of changes in malaria transmission, the peak in malaria morbidity and mortality might shift toward older age groups [21]. Future studies including older age groups are, therefore, needed. Also, entomological and environmental data would be useful to monitor changes in malaria endemicity.

## Conclusion

This study suggests a large decline in malaria prevalence over a 6-year period in a mesoendemic region. However, high disease prevalence remains present in rural villages. This analysis shows also the important role poverty may play as a risk factor for malaria infection, and highlights unexplained heterogeneity in spatial structure as related to malaria prevalence that should be investigated further.

## Competing interests

The authors declare that they have no competing interests.

## Authors' contributions

CN and PDB participated in study design, data collection, statistical analysis, and manuscript drafting. ET participated in data collection, statistical analysis, and manuscript drafting. FG participated in study design, data collection, and manuscript review. YB participated in data collection, data analysis, and manuscript review. BS provided scientific advice for the study design, participated in data collection, and reviewed the manuscript. JFE participated in the study design, provided scientific advice for data analysis and interpretation, and reviewed the manuscript. All authors read and approved the final manuscript.

## Supplementary Material

Additional file 1 **Factors associated with bed-net ownership and use, 2010, n = 941 households**. Click here for file
