# Supplementary material for: Heterogeneous decrease in malaria prevalence in children over a six-year period in south-western Uganda
Source: Malar J. 2011 May 18;10:132. doi: 10.1186/1475-2875-10-132 (PMC3120731; doi:10.1186/1475-2875-10-132)
Supplement: Additional file 1 — Factors associated with bed-net ownership and use, 2010, n = 941 households. [file 1475-2875-10-132-S1.DOC]

## Factors associated with bed-net ownership and use, 2010, n = 941 households

|  | | **Bed-net ownership** | | **Bed-net use by adults** | | **Bed-net use by children <5 years old** | |
| --- | --- | --- | --- | --- | --- | --- | --- |
|  | | **Univariate,  OR (95% CI)** | **Multivariate,  OR (95% CI)** | **Univariate,  OR (95% CI)** | **Multivariate,  OR (95% CI)** | **Univariate,  OR (95% CI)** | **Multivariate,  OR (95% CI)** |
| Residence (urban vs rural) | | 0.39 (0.29-0.52) | 0.74 (0.53-1.03) | 0.72 (0.48-1.09) |  | 0.48 (0.37-0.63) |  |
| Education level^§^ | |  |  |  |  |  |  |
|  | Primary | 1.75 (1.19-2.59) | 1.2 (0.79-1.83) | 1.37 (0.72-2.62) | 1.34 (0.7-2.57) | 2.51 (1.67-3.79) | 1.87 (1.21-2.89) |
|  | Secondary | 4.19 (2.68-6.56) | 1.98 (1.19-3.29) | 1.64 (0.84-3.21) | 1.52 (0.77-2.99) | 5.3 (3.38-8.3) | 3.06 (1.87-4.98) |
|  | ≥ Secondary | 6.71 (3.48-12.95) | 2.42 (1.17-5.01) | 2.48 (1.04-5.91) | 2.39 (1-5.72) | 7.49 (4.15-13.53) | 3.73 (1.96-7.09) |
| Socioeconomic score^#^ | | 1.38 (1.24-1.53) | 1.39 (1.22-1.58) | 1.12 (0.97-1.3) |  | 1.27 (1.15-1.4) | 1.27 (1.13-1.44) |
| Housing score^*^ | | 1.86 (1.52-2.29) |  | 1.13 (0.82-1.57) |  | 1.67 (1.37-2.04) |  |
| Household size | | 0.93 (0.86-0.99) |  | 0.9 (0.81-1) | 0.9 (0.81-1) | 0.90 (0.84-0.96) | 0.9 (0.84-0.97) |
| Mosquito cited as the malaria vector | | 2.59 (1.78-3.76) |  | 1.08 (0.56-2.1) |  | 2.37 (1.62-3.45) |  |
| Bed net cited as a method to prevent malaria | | 3.81 (2.6-5.6) | 2.27 (1.48-3.47) | 1.82 (0.95-3.5) |  | 3.76 (2.51-5.63) | 2.35 (1.52-3.62) |

^§^Highest education level attended by the head of the household (reference category: no education);  ^#^ Standardized score computed using data on household’s ownership of various assets (see details in Method section); *Score computed using the data on household’s walls and roof structure (see details in Method section);
